# Ocean inspiration for a sustainable future: Rivers to Seas engagement and a UN Ocean Agency?

**DOI:** 10.1007/s13280-025-02289-4

**Published:** 2025-10-29

**Authors:** Adam Moolna

**Affiliations:** Keele University, Staffordshire, ST5 5BG UK

**Keywords:** Blue Economy, Ocean literacy, Ocean sustainability, Public engagement, SDGs, Sustainable development

## Abstract

Ocean sustainability matters to everyone because planetary health depends on the ocean and a healthy ocean requires a sustainability transformation across society, land and sea. The ocean is a critical arena for addressing the triple planetary crisis of climate change, biodiversity loss, and pollution. Public engagement with ocean sustainability shows considerable potential to drive forward global sustainability action, as seen in contemporary concerns regarding marine plastic pollution rallying political leaders to develop a global plastics treaty. Despite this success, communities inland can still be much better connected to ocean sustainability and should be engaged to hold decision-makers to account for environmental and social progress alongside political interest in the growing ocean economy. Accessible and emotive messaging is required for public and political audiences, but delivery needs are complex, so we must ensure advances in public engagement are in tandem with translation into appropriate action. This Perspective recommends that we: (1) use a Rivers to Seas paradigm to better connect public support for ocean sustainability with land-based populations; (2) use accessible and emotive public messaging connected to detailed and complex delivery through principle-based approaches; and (3) create a UN Ocean Agency alongside the post-2030 sustainable development agenda to advance the changes needed.

## Introduction

The ocean is emerging as both a focus for growing public engagement with sustainability issues (Shellock et al. 2024) and as a key frontier of expansion in human activities (Jouffray et al. 2020). The public is engaged and passionate about ocean sustainability with, for example, concerns about marine plastic pollution such as entangled sea turtles driving public pressure for progress on global plastics action (Tiller et al. 2019). People think saving turtles and coral reefs is exciting, and they care. There is increasing popular understanding of humanity’s connection with the ocean and such “ocean literacy” is a rising area of research (McKinley et al. 2023). But ocean sustainability needs to improve because ocean health continues to decline (Sumaila et al. 2021). Substantial issues are emerging for how humans use, sometimes abuse, and try to protect our ocean, including our continuing failure to sustainably manage wild-capture marine fisheries (FAO 2024). This is part of a climate change, biodiversity loss, and pollution triple planetary crisis with complex interconnected challenges that need coordinated action to address (UNFCCC 2023). Ocean sustainability depends on wider progress across terrestrial sustainability and global society because the ocean receives much of the impact of our land-sourced issues. Conversely, ocean health affects planetary health including climate, ecosystem services, and disaster risk (Talukder et al. 2022).

We live on an ocean planet—and the ocean surrounds and affects all of us. The ocean covers 71% of Earth’s surface and is dotted with islands of land, the largest of which we call continents. Equitable and sustainable approaches to managing the ocean and the expanding ocean economy are vital for everyone, and we need more effective global public involvement in ocean sustainability. Informed public pressure is needed to shape appropriate aims, energise political attention, and subject ocean governance and policies to scrutiny. That is challenging because, although accessible and emotive messages (such as those using plastic-entangled turtles) are needed for public support and political attention, delivery of sustainable development requires detail and complexity behind the headlines to underpin effective action. We see this, for example, with the way the “30 by 30” public push to protect 30% of our ocean by 2030 has led to a political focus on very large Marine Protected Areas that add substantial coverage to this area-based target but distract from the many much smaller sites in a global network that we really need for a healthy ocean (Jones and de Santo 2016; Pike et al. 2024). Better connected public and political engagement with the complexities and compromises needed for effective ocean sustainability can appropriately shape, as well as energise, improved wider sustainable development frameworks (Claudet et al. 2020).

This Perspective aims to set out an analysis of why public engagement with ocean sustainability matters for a sustainable future and how it can be better connected to global sustainable development. The first section sets out how the ocean is a critical arena for sustainability action that, with public support for ocean sustainability and political interest in the ocean economy, can drive wider societal transformation. The second section explores how ocean sustainability can be better connected to populations living inland, why accessible and emotive public messaging needs to be connected to complex delivery, and how this can be taken forward as we look to develop the post-2030 United Nations (UN) sustainable development agenda. The article concludes with recommendations that we use a Rivers to Seas paradigm for public engagement with ocean sustainability, connect public messaging to complex delivery through principle-based approaches, and create a new UN Ocean Agency to advance these changes.

## The threat to our future and why ocean sustainability matters

### Sustainable development and the ocean as a critical arena for action

We face a worsening climate change, biodiversity loss, and pollution triple planetary crisis with interconnected challenges, including ocean degradation and social inequality, that needs better integrated action (UNFCCC 2023). That is despite more than three decades of coordinated global efforts since the 1992 UN Conference on Environment and Development in Rio de Janeiro, Brazil. The Rio Earth Summit (Rio 92) agreed on sustainable development as a three-pillar approach (Purvis et al. 2019), referring to protection and enhancement of biodiversity and environmental function, social inclusivity of opportunities and livelihoods, and economic prosperity both for itself and to resource the environmental and social pillars. The 17 UN Sustainable Development Goals (SDGs) are our current global framework agreed on in 2015 (Chasek et al. 2016) but progress towards the 169 targets of the SDGs has been partial and slow, with only a few likely to be met by 2030 as intended (Moallemi et al. 2022). Evidence suggests the impact of the SDGs has been largely restricted to political rhetoric, discourse, and governance structures with limited change in legislation or resource allocation (Biermann et al. 2023). High level policies have typically lost coherence with the challenges of translation to real world action and the complexities of operationalising (Coscieme et al. 2021).

Ocean sustainability is integral to wider planetary sustainability. The ocean is substantially affected by global climate change, driven by the fossil fuel and land use of our terrestrial populations, people far inland as much as those close to the coast. A healthy ocean needs nature action across the whole biosphere and global pollution stems from land-based sources. We need better integrative management of the seas with freshwaters (Michels-Brito et al. 2024) because the land surfaces of our planet, even far from the coast, form the catchment basins of rivers that transport land-based sources of pollution such as plastics to the ocean (Rellán et al. 2023). Ocean sustainability is pushing forward land-sea frameworks such as integrated coastal and marine management (Eger et al. 2021) alongside broadscale and cross-sectoral approaches such as marine spatial planning (Santos et al. 2021) and integrated ocean management (Winther et al. 2020).

The ocean is emerging as a critical arena for climate, nature, and pollution action addressing the triple planetary crisis. Climate action, for example, includes carbon capture through coastal and marine ecosystems, renewables, shipping, and climate-friendly aquaculture (Hoegh-Guldberg et al. 2019). The ocean also offers major potential for economic growth aligned with addressing climate change, food security, and biodiversity (Lubchenco et al. 2020). Human activities focused on the ocean are rapidly expanding with growing political, economic, scientific, and cultural interest in what has been termed the “Blue Acceleration” (Jouffray et al. 2020). Concepts for ocean-focused sustainable development are generally referred to as (equitable and sustainable) “Blue Economy” approaches. But these often have questionable alignment with three-pillar principles of sustainable development (Voyer et al. 2018); and substantial geopolitical and social justice concerns have emerged (Bennett et al. 2023). Social justice concerns are exacerbated in ocean sustainability because of rapid economic growth and expansion into new sectors, because national ocean waters are typically the property of the state without private ownership rights as on land, and because of inadequate governance frameworks for emerging sectors in the high seas.

Ocean degradation is worsening as expanding patterns of ocean resource use threaten the natural capital and health of the ocean on which long-term sustainable growth depends (Sumaila et al. 2021). There is, for example, alarming global data on the increasing scale of overfishing—the percentage of marine fisheries harvested at unsustainable levels has increased steadily from the mid-1970s and continues to increase (FAO 2024). Our approach to managing the ocean is not keeping up with the rapidly expanding activities, intensity of use, and geographical areas we are impacting. Wild capture marine fisheries are changing and expanding into new depths and areas, new species and populations, to which ecosystem-based approaches and governance systems for the global marine fisheries commons need to adapt (Paniagua and Rayamajhee 2024). Marine aquaculture is expanding with changing uses, growing intensity, and new approaches that challenge our abilities to manage in ecologically sound and socially equitable ways (Campbell et al. 2021). Mining of deep-sea mineral resources, including metallic nodules largely found in the high seas, is potentially on the brink of substantial commercialisation (Ardito and Rovere 2022). The high seas especially have multiple and substantial knowledge gaps, from social and political governance to ecology and fisheries, that need advances for better management (Jarvis and Young 2023).

### Engagement with the ocean can incentivise and advance global progress

The ocean captures public attention that drives societal pressure to do better and inspires change in how we protect our environment. We are seeing this, for example, with progress on a global plastics treaty largely inspired by marine plastic pollution (Tiller et al. 2019). That is despite estimates that 98.5% of plastic waste remains in terrestrial environments and largely pollutes inland freshwaters (Meijer et al. 2021). The high profile of ocean plastic pollution in the public imagination drives action that has its main impact on land. The public is engaged with ocean sustainability and passionate. This matters because that engagement drives societal pressure on governments, on multinational bodies, and on businesses to do better. Claudet et al. (2020) suggested we use the political attention of the UN Decade for Ocean Science 2020–30 to establish ocean science as a key foundation for broader sustainability transformations by identifying appropriate policy levers and improving science-policy interfaces, ocean-climate finance systems, and partnerships. The human right to a healthy ocean was recognised by the UN General Assembly in 2022—but Bennett et al. (2024) warn that such political rhetoric needs to be backed up by real action.

International cooperation is critical to addressing regional and global environmental issues and depends on public engagement, political consensus, and the advocacy of scientists—as seen in success on European regional acid rain (Grennfelt et al. 2020) and on removing ozone depleting products from the global economy with the Montreal Protocol (Gonzalez et al. 2015). Ocean spaces, whether international or national in jurisdiction, are highly interconnected and cooperation between states is crucial. Ocean circulation, marine ecological expanses, and human influences transcend the administrative borders we mark on our maps. There are various overlapping regional and subregional groupings in ocean governance, including fisheries management organisations and bodies for biodiversity and pollution approaches (Mahon and Fanning 2019). The Regional Seas Programmes established from the 1970s by the UN Environment Programme (UNEP) support collaborative multi-sectoral management around the world. But international cooperation and the application of ocean science to ocean sustainability needs to improve.

Political interest in the ocean is driven by rising economic opportunities and by geopolitical considerations. These have led to attempts to exert increased control of ocean space including claims to enhanced rights or extended jurisdictions under the UN Convention on the Law of the Sea (UNCLOS). Existing and new economic activities are projected to increasingly expand into the high seas (Jouffray et al. 2020), or Areas Beyond National Jurisdiction, that account for two-thirds of the ocean by area and have shared rights of use and exploitation for all nation states under UNCLOS. Concerns for the environmental impacts of expanding high seas activities and how these are governed has led to the Biological diversity in areas Beyond National Jurisdiction (BBNJ) treaty (Kim 2024). But the high seas are at risk of unilateral activities from nation states outside of UNCLOS, most notably the USA and potential deep-sea mining (Willaert 2021), and there have been growing efforts to effectively territorialise areas of the high seas (Lambach 2021).

Public pressure will be key to restraining political leaders and fighting against the divisions that threaten the collaborative approaches needed for ocean sustainability. But how can we better bring together political interest in the ocean economy and scientific advances in management with public concerns for the ocean environment and pressure for action? For most of the global population living inland, present engagement with ocean sustainability is from an outside perspective and with symbolic high-profile issues such as sea turtles, coral reefs, oil spills, and marine plastic pollution. This needs mission-oriented translation and reframing into locally relevant approaches (Uyarra et al. 2025).

## Better engaging public and political interest in ocean sustainability

### Connecting with land-based societies using a “Rivers to Seas” paradigm

“Ridge to Reef” has emerged as a catchy term for land-sea integrated approaches to protect coral reefs from land-based sources of pollution and sediment impacts (Carlson et al. 2019). Can we extend this with a “Rivers to Seas” paradigm? Wherever people live, they inhabit part of a river catchment that (almost always) leads to the ocean. Manaus in Brazil is 1500 km from the coast, for example, but on a tributary of the Amazon. Minneapolis in the USA is 1600 km from the Atlantic but connected to the ocean by the Mississippi. Civilisation developed along major river systems and rivers play a prominent role in cultural sense of place and belonging, often with evocative names or metaphorical descriptions. Consider the Holy Ganges through India, the Yellow River of China, and the Nile being the lifeblood of Egypt. We can use place-based approaches (Silbernagel et al. 2015) and cultural alignment with such river systems to connect inland populations (and their support for symbolic ocean sustainability issues) with awareness of how rivers and land where they live impact connected coasts and their own ocean “neighbourhoods” (Vincent 2011).

“Rivers to Seas” offers an evocative framework to enable societal pressure for action at the integrated river, coast, and ocean management scales needed (Levin et al. 2020). Operationalising such integrated land-sea public engagement and governance needs education campaigns, harmonisation of regulatory and policy reforms, and development of aligned financial support mechanisms. Public engagement for “Rivers to Seas” can build on the community involvement that is already part of catchment-wide river basin management around the world (Vall-Casas et al. 2024). We should explore what prominent issues and flagship species and habitats can be used as rallying points (Jarić et al. 2025) to meaningfully connect the public support of inland populations for symbolic issues with actionable ocean sustainability. Prominent issues relevant worldwide include flood risk and coastal erosion linked to climate change and sea level rise (Carter et al. 2024). Flagship species might include fish that migrate between rivers and seas and face threats in both, such as eels (Righton et al. 2021). Flagship habitats could be those that are highly affected by river flows and sediment, play an important role in coastal protection and ecosystem services, and have rich and intriguing biodiversity, such as mangroves (Dahdouh-Guebas et al. 2020). Coastal seagrass meadows are similarly linked to coasts and have extensive worldwide public interest in protection and restoration (Jones et al. 2018). But it is only via meaningful dialogue with people across society that we will discover what truly inspires the public discourse.

### Accessible and emotive public messaging linked to detailed and complex delivery

Societal engagement needs publicly and politically accessible and emotive messaging. But we risk not achieving appropriate impact if we do not connect accessible and emotive aims with complex delivery needs. We need to think carefully about what rallying calls such as “30 by 30” (to protect 30% of global land and ocean by 2030) need for effective and appropriate implementation. Public attention also needs to be connected to systemic change to have successful impact—as when marine plastic pollution shown in the BBC (British Broadcasting Corporation) "Blue Planet" documentary series triggered public pressure for regulatory address, that the United Kingdom government then enacted for plastic bags (Males and Van Aelst 2021). Without actionable calls for specific systemic interventions, meaningful change is not embedded—as is argued to be why the annual “Earth Hour” (symbolically switching off non-essential electric lights) has had negligible impact on either long-term behavioural change or climate action (Kountouris 2022).

“30 by 30” has helped drive political momentum for action that embeds systemic change—with, for example, steps taken towards the first Marine Protected Areas (MPAs) in the high seas under the BBNJ Treaty (Kim 2024). Unfortunately, area-based targets have led to a political focus on very large MPAs (VLMPAs) at the expense of distracting attention from small and highly impacted areas (Jones and de Santo 2016). VLMPAs do protect critical hotspots and key ecological refuges but are often in low human impact areas of the remote ocean—and the vast areas added to the 30% target overstates their functional contribution. MPAs are most needed in areas of high human impact and areas of high biodiversity and function, which tend to be around coasts. We need a coherent network of MPAs across our ocean that encompasses complex patterns of biodiversity, ecological interactions, ocean productivity, and human impact (Pike et al. 2024). VLMPAs need to go hand in hand with a network of many smaller ones in and around coasts and areas of high human impact.

MPAs exemplify the complex considerations of sustainability science, including uncertainties of evidence, standards, goals, and interpretation. The total area covered by MPAs is a proxy for ocean protection but not an appropriate metric for a healthy ocean because it does not equate to the quality of ocean-scale ecological protection and health. What indicators might be more consistent with the conservation and restoration of biodiversity and ocean health? We need to explore alternatives better aligned to appropriate ecological scales and parameters, which might be of connectivity, ecological resilience, and productivity. We then, however, face challenges in deciding metrics for indicators and collecting data, how we weigh the relative importance and interactions of multiple metrics, and how we translate these into accessible and emotive public and political messaging. Beyond MPAs and ocean health, we also need more appropriate indicators for sustainable development. Economic growth and Gross Domestic Product remain the main metrics used to measure the productivity and development of countries, although combining the UN Human Development Index with environmental and sustainability indicators is emerging as an alternative (Hickel 2020). Complex and subjective environmental and social parameters are more challenging to value than economic productivity, which is readily quantified in monetary terms. Perhaps because of this, environmental stewardship and social fairness considerations have in practice remained subsidiary to, rather than synergized with, a continuing focus on economic development (Eisenmeger et al. 2020).

Ocean sustainability is a wicked problem (Salas et al. 2022) with uncertain definitions and potential solutions that are contested by stakeholders across a varied knowledge and power spectrum. Delivery has complex synergistic, antagonistic, and additive interactions with often competing impacts. These also need to be considered at multiple spatial scales aligned to ocean function that do not readily align with political and economic geographic jurisdictions. Attempts at quantification and choice of appropriate metrics for cost–benefit analysis are therefore inevitably contested, typically too complex and economically biased, and ineffective at providing “objective” data to appropriately inform decision-making and trade-offs across the spatial (and stakeholder) scales necessary. We need coherence across environmental, social, and economic aims; and national approaches coordinated within regional strategic frameworks. This mean not getting lost in headline economic growth, or area-based targets for MPAs, and instead aligning decision-making to the principles of a healthy resilient planet supporting a fair and socially inclusive global society—supported by metrics as context appropriate. We could use, for example, environmental and social impact assessment approaches revised to support fast, instinctive, principled decision-making as proposed by Bond et al. (2024). Social equity especially is inherently complex, and principles need to be conceptualised in “an accessible and simple form” as proposed by Croft et al. (2024) for participatory governance. Principles facilitate the accessible and emotive messaging for public and political engagement that is how we will drive forward meaningful action.

### Advancing the changes needed through a new UN Ocean Agency?

Recognising the importance of ocean diplomacy (Polejack 2021), political leaders should consider the creation of a UN Ocean Agency alongside the post-2030 UN sustainable development agenda to advance the changes needed. This could lead and coordinate the responsibilities currently dispersed across various bodies and bring together nation states within UNCLOS and those that remain outside. Uniting marine responsibilities siloed in different UN bodies in an International Ocean Agency had previously been discussed in the 1980s (Grip 2017). A UN Ocean Agency could build upon political engagement platforms and entities that bridge the science-policy divide, such as the High Level Panel for a Sustainable Ocean Economy, to inspire and facilitate cooperation, to shine a light on emerging best practices, and to support worldwide uptake and coordination (Claudet et al. 2020; Lubchenco et al. 2020). It could provide the melting pot within which politicians, scientists, activists, artists, and wider contributors develop the shared understandings, ambitions, and ways of collaborative working that we need.

This UN Ocean Agency could also develop frameworks to support operationalisation of the post-2030 goals at a regional level, drawing on existing models such as UNEP’s Regional Seas programmes. These could facilitate shared funding, management, and expertise across functionally appropriate regional domains. Distribution of shared funding to where it is needed could build on existing models such as the European Union’s regional development funds (Spilioti and Anastasiou 2024). A UN Ocean Agency should also act to shape investment frameworks aligned to sustainable development, and energise these with public funding, to attract the much greater sums needed—and that only private finance will be able to supply. Economists estimate we need additional investments of trillions of US dollars annually to meet the 17 SDGs, with SDG14 "Life below water" alone needing an extra $150bn per year (Kulkarni et al. 2022).

## Conclusion and recommendations

Alongside political interest in the growing ocean economy, ocean sustainability is emerging as a key focus of public pressure for action on climate change, biodiversity loss, and pollution. The ocean inspires, and ocean sustainability can drive forward a step-change in coordinated global action towards a prosperous, fair, and environmentally sustainable future. To better integrate public support for ocean sustainability with sustainable development needs for both the ocean and wider planet, this Perspective recommends that we:

1. Use a Rivers to Seas paradigm to better connect public support for ocean sustainability with land-based populations.
2. Use accessible and emotive public messaging connected to detailed and complex delivery through principle-based approaches.
3. Create a UN Ocean Agency alongside the post-2030 sustainable development agenda to advance the changes needed
